# Dual-energy CT and ceramic or titanium prostheses material reduce CT artifacts and provide superior image quality of total knee arthroplasty

**DOI:** 10.1007/s00167-018-5001-8

**Published:** 2018-06-07

**Authors:** Maximilian F. Kasparek, Michael Töpker, Mathias Lazar, Michael Weber, Michael Kasparek, Thomas Mang, Paul Apfaltrer, Bernd Kubista, Reinhard Windhager, Helmut Ringl

**Affiliations:** 1 Department of Orthopaedics and Trauma Surgery, Vienna General Hospital, Medical University of Vienna, Waehringer Guertel 18-20, 1090 Vienna, Austria; 2 Department of Biomedical Imaging and Image-Guided Therapy, Medical University of Vienna, Waehringer Guertel 18-20, 1090 Vienna, Austria; 3 Department of Orthopaedic Surgery, Orthopaedic Hospital Speising, Speisinger Str. 109, 1130 Vienna, Austria

**Keywords:** Total knee arthroplasty, Single-energy CT, Dual-energy CT, Blooming artifacts, Streak artifacts, Mono-energetic imaging

## Abstract

**Purpose:**

To evaluate the influence of different scan parameters for single-energy CT and dual-energy CT, as well as the impact of different material used in a TKA prosthesis on image quality and the extent of metal artifacts.

**Methods:**

Eight pairs of TKA prostheses from different vendors were examined in a phantom set-up. Each pair consisted of a conventional CoCr prosthesis and the corresponding anti-allergic prosthesis (full titanium, ceramic, or ceramic-coated) from the same vendor. Nine different (seven dual-energy CT and two single-energy CT) scan protocols with different characteristics were used to determine the most suitable CT protocol for TKA imaging. Quantitative image analysis included assessment of blooming artifacts (metal implants appear thicker on CT than they are, given as virtual growth in mm in this paper) and streak artifacts (thick dark lines around metal). Qualitative image analysis was used to investigate the bone–prosthesis interface.

**Results:**

The full titanium prosthesis and full ceramic knee showed significantly fewer blooming artifacts compared to the standard CoCr prosthesis (mean virtual growth 0.6–2.2 mm compared to 2.9–4.6 mm, *p* < 0.001). Dual-energy CT protocols showed less blooming (range 3.3–3.8 mm) compared to single-energy protocols (4.6–5.5 mm). The full titanium and full ceramic prostheses showed significantly fewer streak artifacts (mean standard deviation 77–86 Hounsfield unit (HU)) compared to the standard CoCr prosthesis (277–334 HU, *p* < 0.001). All dual-energy CT protocols had fewer metal streak artifacts (215–296 HU compared to single-energy CT protocols (392–497 HU)). Full titanium and ceramic prostheses were ranked superior with regard to the image quality at the bone/prosthesis interface compared to a standard CoCr prosthesis, and all dual-energy CT protocols were ranked better than single-energy protocols.

**Conclusions:**

Dual-energy CT and ceramic or titanium prostheses reduce CT artifacts and provide superior image quality of total knee arthroplasty at the bone/prosthesis interface. These findings support the use of dual-energy CT as a solid imaging base for clinical decision-making and the use of full-titanium or ceramic prostheses to allow for better CT visualization of the bone–prosthesis interface.

**Electronic supplementary material:**

The online version of this article (10.1007/s00167-018-5001-8) contains supplementary material, which is available to authorized users.

## Introduction

Common complications of TKA are loosening of the prosthesis or infection [3, 4, 18]. Postoperative care of TKAs usually includes periodic physical examinations, as well as radiographic follow-up [17]. In the presence of abnormal findings in postoperative follow-up examinations, e.g., suspicious loosening or osteolytic lesions, further work-up includes scintigraphy [20] and computed tomography (CT) [10, 19]. The latter provides excellent spatial resolution of the bones, soft-tissue, and prosthetic material, but it has some limitations due to metal artifacts at the bone/prosthesis interface [25].

**Fig. 1:**
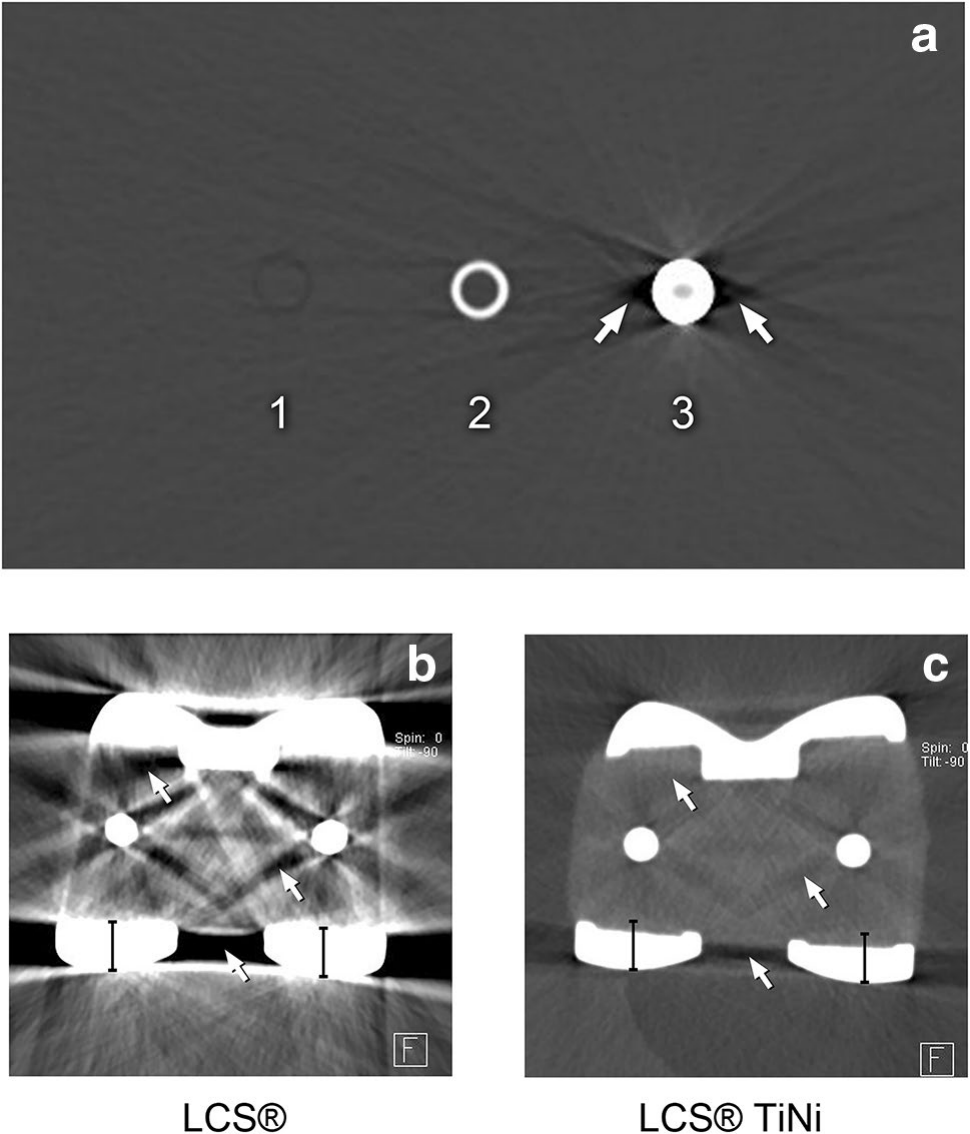
CT scan of a water phantom showing blooming and beam-hardening artifacts (**a**). The scan shows a section through a plastic tube (1, low attenuation), an aluminum tube (2, medium attenuation), and a steel tube (3, high attenuation). All tubes had the same diameter of 10 mm and a wall thickness of 1 mm. The plastic tube is less dense than water, and was, therefore, visible as a dark ring without virtual thickening of the wall. The aluminum tube is denser, and was, therefore, visualized as a bright ring, with the wall thickness slightly thicker than the actual thickness, representing a slight blooming artifact. The steel tube, however, has the highest attenuation of all three materials and showed, therefore, a pronounced blooming artifact with a wall thickness four times larger than its physical size. The dark streaks and triangles (white arrows) around the steel pipe are typical streak artifacts that arise from very dense materials, such as steel. A comparison between a conventional CoCr steel alloy prosthesis (**b**) and a titanium prosthesis (**c**). White arrows show considerable streak artifacts in close proximity to the metal in the steel prosthesis, while the titanium prosthesis offered only a low amount of artifacts. The magnitude of the blooming artifacts for steel is demonstrated by the superimposed calipers (same length) on the condyles of both prostheses

The two major artifacts that appear on CT around metallic implants are streak and blooming artifacts (Fig. 1). Several methods have been proposed to reduce metal artifacts [6, 12, 16, 24, 26], which are the result of photon starvation and beam-hardening of the metal components of the prosthesis [2]. These methods include changes in scan parameters, such as higher tube voltage, and the use of image post-processing algorithms [13, 14, 23].

In addition to these methods, dual-energy CT has been proven to reduce metal artifacts, using both a hardware and software approach for the best image quality [1, 7, 27]. Dual-energy CT scanners simultaneously acquire two CT datasets with different X-ray tube voltages, usually 80 and 140 kV. The images derived from the 80 kV tube voltage are best suited for visualization of soft tissue, whereas the images from the 140 kV tube are best for assessment of bones and metal-implant interfaces. From these two datasets, image post-processing allows for calculation of virtual mono-energetic images, which further reduces metal artifacts [11]. Dual-energy CT scans are dose-neutral compared to a standard single-energy CT [1, 9].

In addition, the amount of metal artifacts is highly dependent on the material of the prosthesis [8, 14]. Almost all vendors of knee prostheses provide a standard prosthesis that consists of Cobalt-Chromium (CoCr), as well an additional model for patients with a history of allergy to nickel, which are made of titanium or ceramic or are coated with a ceramic alloy or an oxidized zirconium alloy. Based on clinical experience, large differences in the extent of metal artifacts have been observed between different TKA models and vendors.

The use of different CT protocols, including dual-energy CT imaging in TKA, has not been assessed in the literature, nor do any published data exist about the influence of the material composition of TKA on CT.

In this study, therefore, the influence of different scan parameters, using single-energy CT and dual-energy CT, as well as the impact of different materials used in the TKA prosthesis on image quality and on the extent of metal artifacts, was assessed. The aim of this study was to provide a better understanding of these artifacts, to determine the best-suited CT protocol, and to obtain a comprehensive view of the CT image quality of different TKA material compositions.

## Materials and methods

For this study, the femoral components of eight pairs of TKA prostheses from different vendors were examined in a phantom setup (Table 1). Each pair consisted of a conventional CoCr prosthesis and the corresponding anti-allergic prosthesis from the same vendor. The non-CoCr prostheses consisted of either full titanium, a ceramic alloy material, a ceramic-coated CoCr prosthesis, or an oxidized zirconium alloy. There is no standardized size specification for prostheses between the different vendors and even the proportions of different parts of the prostheses varied considerably between the vendors. Because of these differences in size and construction, in this study, only the extent of artifacts within a particular vendor pair was compared, with regard to the impact of different material compositions on the images. Within all pairs, the actual diameters of the condyles and the shapes were always identical.

**Table 1:** All prosthesis pairs and material characteristics of all vendors

List of total knee arthroplasty implants (TKA)				
Pairs	Standard model	Material	“Anti-allergic” model	Material
1	LCS^®^	CoCr-alloy	LCS^®^ TiNi	Titanium alloy prosthesis with a titanium nitride ceramic surface
2	ACS^®^	CoCrMo-alloy	ACS^®^ Titannitrid (TiN)	CoCr prosthesis coated with titanium nitride
3	Vanguard™	CoCr-alloy	Vanguard™ Titan-Niob-Nitrid (TiNbN)	CoCr prosthesis coated with titanium-niobium-nitride
4	Columbus^®^	CoCr-alloy	AS Columbus^®^	CoCr prosthesis ceramic coated with zirconium and zirconium nitride
5	Legion™	CoCr-alloy	Legion™ Oxinium^®^	Zirconium alloy prosthesis with an oxidized ceramic surface
6	NexGen^®^ LPS	CoCrMo-alloy	NexGen^®^ Tivanium^®^ LPS	Full titanium alloy prosthesis
7	BPK-S	CoCr-alloy	BPK-S ceramic knee	Ceramic prosthesis
8	Scorpio^®^	CoCr-alloy	Scorpio^®^ TiNi	CoCr prosthesis coated with titanium-niobium-oxynitride

1. DePuy Synthes Inc., Warsaw, IN, USA; 2. Implantcast Gmbh, Buxtehude, Germany; 3. Biomet Inc., Warsaw, IN, USA; 4. Aesculap AG, Tuttlingen, Germany; 5. Smith & Nephew PLC, London, UK; 6. Zimmer Inc., Warsaw, USA; 7. Peter Brehm Gmbh, Weisendorf, Germany/CeramTec Gmbh, Plochingen, Germany; 8. Stryker Corporation, MI, Kalamazoo, USA

**Fig. 2:**
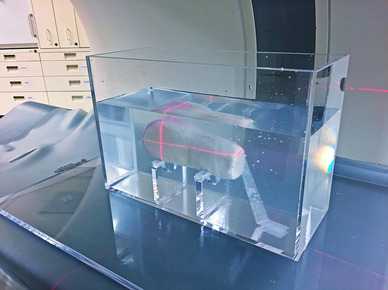
Play Doh^®^ prosthetic phantom setting. For simulation of the anatomic conditions, the distal femoral shaft of each TKA was formed with Play Doh^®^ as a surrogate for cancellous bone. Play Doh^®^ allowed the simulation of cancellous bone with comparable attenuation Hounsfield values of around 260 HU. The phantom was placed underwater in an acrylic glass water tank to simulate soft tissue around the knee joint and to avoid inappropriate artifacts

For simulation of the periprosthetic anatomic conditions and their proper attenuation characteristics, we used Play Doh^®^ (Hasbro, Pawtucket, Rhode Island, USA) as a surrogate for cancellous bone. Both materials offer similar attenuation Hounsfield values of around 260 Hounsfield units (HU). For each prosthesis, the distal femoral shaft was formed with Play Doh^®^. The phantom setup is shown in Fig. 2.

### CT scanning protocols

All scans were performed with a second-generation, dual-energy, multi-detector CT scanner (Siemens Somatom Definition Flash CT, Siemens Healthineers, Forchheim, Germany). In total, nine different protocols were applied to 16 different phantoms, resulting in 144 scans. Seven scans were performed with a dual-energy CT technique, ranging from 80 kV/Sn 140 kV (tube A/tube B) to 100 kV/Sn 140 kV, and two scans with single-energy CT (120 and 140 kV). Dose modulation was active for protocols 1–4, and was deactivated for protocols 5–9. In addition, the reference mAs and the mAs were changed for the different protocols. All scans were reconstructed in a transverse orientation at a slice thickness of 0.6 mm and an increment of 0.4 mm. Exact parameters for each protocol are given in Table 2. The dual-energy CT datasets were post-processed and mono-energetic images were reconstructed at a level of 105 keV, as recommended by Bamberg et al. [1].

**Table 2:** Scan and dose parameters for all protocols, including the subjective ranking of image quality at the bone/prosthesis interface

Number of scan protocols	Dual (DE)- or single-energy (SE) CT	Tube A (kV)	Tube B (kV)	CARE DOSE	Ref. mAs^a^		Pitch	Rotation time (s)	Kernel	Iterative reconstruction SAFIRE strength	Dose (CTDIvol)	Blooming artifacts: mean virtual growth mm (± SD)	Streak artifacts ROI: mean image noise HU (± SD)	Subjective image quality ranking^b^
1	DE	100	140	On	200	155	0.8	0.5	Q40f	2	4.0	3.4 (± 1.6)	258.0 (± 139.1)	5
2	DE	100	140	On	300	232	0.6	0.5	Q40f	2	6.0	3.4 (± 1.6)	242.8 (± 135.4)	4
3	DE	100	140	On	100	77	0.6	0.5	Q40f	2	2.0	3.8 (± 1.9)	296.3 (± 127.9)	6
4	DE	80	140	On	401	155	0.8	0.5	Q40f	2	3.7	3.4 (± 1.7)	256.9 (± 134.6)	7
5	DE	100	140	Off	150	116	0.4	0.5	Q40f	2	11.8	3.3 (± 1.6)	237.7 (± 138.4)	3
6	DE	100	140	Off	200	155	0.4	0.5	Q40f	2	15.7	3.4 (± 1.6)	225.1 (± 141.4)	2
7	DE	100	140	Off	250	193	0.4	0.5	Q40f	2	19.6	3.4 (± 1.6)	214.8 (± 139.0)	1
8	SE	120	–	Off	210	–	0.6	0.5	I40f	2	14.2	5.5 (± 2.1)	497.2 (± 208.1)	8
9	SE	140	–	Off	210	–	0.6	0.5	I40f	2	20.6	4.6 (± 1.9)	392.4 (± 172.3)	9

Blooming artifacts are given as virtual growth (the difference between the actual diameter and the CT measurement) as the mean of all measured prostheses; streak artifacts are given as mean image noise for the mean of all prostheses. For both blooming and streak measurements, higher numbers represent more artifacts, and, therefore, lower image quality

*mm* millimeter, *HU* Hounsfield unit

^a^Ref. mAs (quality reference milliampere × second) represents the value suitable for an average-sized patient that is automatically adjusted to the required value by the scanner according to the actual size of the patient

^b^Consensus ranking (1–9, 1 indicating the best result)

### Quantitative image analysis

The two major artifacts that were assessed in this study are streak and blooming artifacts. Streak artifacts can be recognized as thick, dark lines that arise from metal implants due to beam-hardening and photon starvation effects [2] (Fig. 1). In our phantom, we placed eight regions of interest (ROI) in close vicinity to the prosthesis at standardized positions. Details about the ROI measurements are given in the Supplementary Material.

The second artifact that is present in patients with metal implants on computed tomography is a blooming artifact [2, 5, 22] (Fig. 1). Due to partial volume and beam-hardening effects, objects with very high attenuation, such as metal implants, are displayed larger than their physical size in computed tomography [22]. This effect is dependent on the size and the attenuation of the object and the scan parameters. The difference between the actual physical size of the condyles of the prosthesis and the size measured on CT is further addressed as virtual growth and represents the extent of the blooming artifact. For instance, if the actual physical thickness is 6 mm and the measured size is 9 mm, the virtual growth would be 3 mm (because of multiple measurements, the SD is given in parentheses). Blooming artifacts were assessed by two readers (one advanced radiology resident, and one board-certified radiologist, with 4 and 11 years of experience in reading CT, respectively). They independently measured the maximum thickness of the posterior condyle (mm) of the femoral part of the prosthesis with the caliper tool of the PACS (Agfa HealthCare, Mortsel, Belgium). Further details about this reading process are given in the Supplementary Material section.

### Qualitative image analysis

For discrimination of differences in image quality between certain protocols, a forced-choice sorting comparison algorithm was chosen. The same readers were asked independently of each other to sort the images that resulted from the nine different protocols per prosthesis according to the image quality at the cancellous bone/prosthesis interface. In addition to the sorting, the image quality at the metal–bone-interface was assessed on a five-point Likert scale (1: very good image quality; 2: good; 3: intermediate; 4: poor; 5: very poor image quality).

For the current study, an institutional review board approval was not required at our institution.

### Statistical analysis

Statistical planning and analysis for this study was performed by a professional statistician, using IBM SPSS Statistics for Windows (Version 23; IBM). Metric variables are described by means and standard deviations, if normally distributed. The extent of blooming artifacts and streak artifacts within each particular vendor pair was compared using paired *t* tests. Differences between scan protocols were tested using one-way ANOVA and Bonferroni-corrected post hoc tests. For the image quality and comparison between each prosthesis pair, the median of the Likert scale for both readers was used. Inter-rater and intra-rater agreement were calculated using the intraclass correlation coefficient (ICC). For blooming artifact measurements, inter-rater and intra-rater ICCs were 0.907 and 0.961, respectively. For the assessment of image quality at the bone/prosthesis interface, inter-rater and intra-rater ICCs were 0.942 and 0.963, respectively. For multiple comparisons of the scan protocols, means were compared using the Bonferroni method. A *p* value < 0.05 was considered statistically significant. In this study, very large effect sizes, with an epsilon up to 2.3, were observed. A post hoc power calculation revealed that, using nine protocols, the power to detect the observed differences is up to 95% for image noise (given an alpha of 5% two-sided).

## Results

### Blooming artifacts (virtual growth)

#### Influence of material

The highest virtual growth was observed for the Legion™ prosthesis (7.4 mm) and the lowest was found for the LCS^®^ Titan prosthesis, with only 0.6 mm. We found significantly less virtual growth for the following anti-allergic models compared to the standard CoCr models: LCS^®^ (4.0 mm (*p* < 0.001)); ACS^®^ (0.3 mm (*p* = 0.019)); Legion™ (1.3 mm (*p* < 0.001)); NexGen^®^ LPS (1.7 mm (*p* < 0.001)); and BPK-S (1.4 mm (*p* < 0.001)). In the Stryker pair (0.4 mm (*p* = 0.001))and the Biomet pair (0.1 mm (n.s.)), a slight increase of the blooming artifact for the anti-allergic models was observed (Table 3).

**Table 3:** Blooming artifacts (virtual growth) and standard deviation of HU (streak artifacts), with the mean of the standard deviation of all measurements near each prosthesi*s* compared for each single pair

Measurement	Blooming artifacts				Streak artifacts			Image quality
Prosthesis	Real diameter^a^ (mm)	Mean CT diameter^a^ (mm)	Blooming: mean virtual growth (mm)	*p* value (paired *t* test)	Mean standard deviation (HU)	Difference	*p* value (paired *t* test)	Median ranking^b^
LCS^®^	8.7	13.3 (± 0.9)	4.6 (± 0.9)	< 0.001	334.3 (± 88.9)	257.1	< 0.001	3
LCS^®^ TiNi	8.7	9.3 (± 0.4)	0.6 (± 0.4)		77.2 (± 40.7)			1
ACS^®^	7.5	12.5 (± 1.3)	5.0 (± 1.3)	0.019	310.3 (± 93.9)	39.4	0.001	2
ACS^®^ Titannitrid (TiN)	7.5	12.2 (± 1.1)	4.7 (± 1.1)		270.9 (± 79.5)			3
Vanguard™	9	12.8 (± 1.0)	3.8 (± 1.0)	n.s.	319.1 (± 113.0)	9.5	n.s.	2
Vanguard™ Titan-Niob-Nitrid (TiNbN)	9	12.9 (± 0.8)	3.9 (± 0.8)		309.6 (± 98.3)			2.5
Columbus^®^	7.4	10.4 (± 0.9)	3.0 (± 0.9)	n.s.	279.1 (± 99.2)	17.7	n.s.	2
AS Columbus^®^	7.4	10.1 (± 0.5)	2.7 (± 0.5)		296.8 (± 124.0)			3
Legion™	11.6	19.0 (± 0.7)	7.4 (± 0.7)	< 0.001	315.3 (± 71.9)	416.0	< 0.001	4
Legion™ Oxinium^®^	11.6	17.7 (± 0.3)	6.1 (± 0.3)		731.3 (± 88.5)			5
NexGen^®^ LPS	11.6	14.5 (± 1.0)	2.9 (± 1.0)	< 0.001	288.3 (± 73.9)	208.8	< 0.001	5
NexGen^®^ Tivanium^®^ LPS	11.6	12.8 (± 0.3)	1.2 (± 0.3)		79.5 (± 27.7)			1
BPK-S	6.1	9.7 (± 0.4)	3.6 (± 0.4)	< 0.001	277.2 (± 121.3)	191.2	< 0.001	4
BPK-S ceramic knee	6.1	8.3 (± 0.2)	2.2 (± 0.2)		86.0 (± 34.5)			1
Scorpio^®^	7.7	12.1 (± 1.0)	4.4 (± 1.0)	0.001	358.5 (± 136.7)	31.9	0.003	3
Scorpio^®^ Titan	7.7	12.5 (± 1.1)	4.8 (± 1.1)		326.6 (± 155.2)			3.5

*mm* millimeter, *HU* Hounsfield unit

^a^Diameter, maximum thickness of the posterior condyles

^b^Five-point Likert scale (1–5, 1 indicating the best result)

#### Influence of protocols

The scan protocol with the lowest virtual growth was observed for scan protocol 5 (dual-energy CT), with a mean of 3.3 mm over all prostheses. The highest virtual growth was observed in the single-energy CT scan protocol 8, with 5.5 mm, which was significantly higher compared to all the other protocols (*p* value ranging from 0.005 to 0.032). Moreover, all dual-energy CT protocols, except protocols 3 (n.s.) and 5 (n.s.), had significantly less blooming artifacts than the single-energy protocol 9 (*p* value 0.018–0.043) (Table 2**)**.

### Streak artifacts

#### Influence of material

**Fig. 3:**
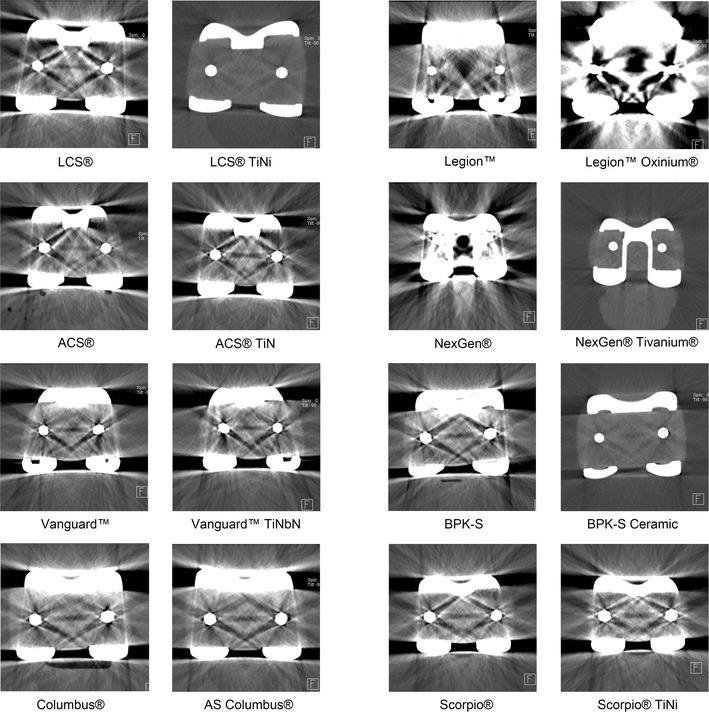
CT images of all vendor prostheses pairs showing the extent of the artifacts. For all images in this gallery, scan protocol 7 (dual-energy CT with mono-energetic imaging at 105 keV) was used. It provided the lowest amount of streak artifacts of all protocols

The full titanium prosthesis LCS^®^ TiNi^®^, the NexGen^®^ Tivanium^®^, and the BPK-S full ceramic knee showed significantly fewer streak artifacts compared to the standard CoCr version (*p* < 0.001). The ACS^®^ and Scorpio^®^ ceramic-coated prosthesis presented with slightly fewer artifacts compared to the standard CoCr prosthesis counterpart (ACS^®^*p* = 0.001, Scorpio^®^*p* = 0.003). However, the Legion™ prosthesis pair showed an inverse distribution, with the Oxinium^®^ version producing a significantly higher number of artifacts compared to the standard model made of CoCr alloy (*p* = 0.001). The Vanguard™ (n.s.) and Columbus^®^ (n.s.) pair showed a comparable number of streak artifacts (Table 3; Fig. 3).

#### Influence of protocols

**Fig. 4:**
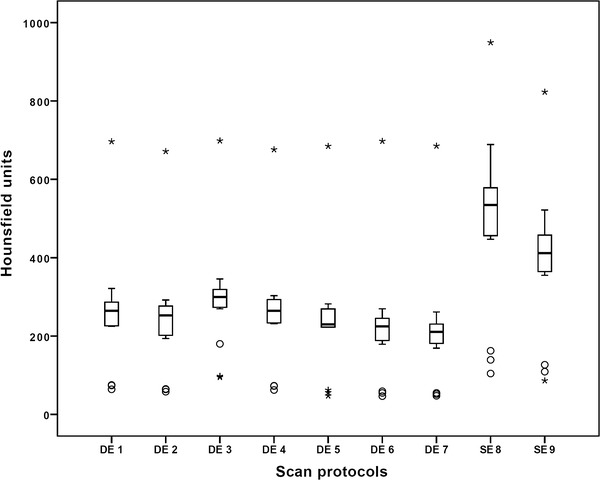
Streak artifacts for all nine scan protocols as a mean over all prostheses: the fewest streak artifacts were observed for scan protocol 7. Dual-energy CT scan protocols (DE 1–7) offered fewer streak artifacts compared to single-energy CT protocols (SE 8 and 9)

The lowest number of streak artifacts was observed for the dual-energy CT protocol 7 without dose modulation, with a mean of standard deviation of 214.8 HU (mean over all prostheses). Except for the dual-energy CT protocol 3 (compared to protocol 9 (n.s.)), all dual-energy CT protocols had significantly fewer streak artifacts (*p* value ranging between 0.001 and 0.014) (Table 2; Fig. 4).

The radiation dose of the dual-energy CT protocols (scan protocols 1–4) was three to four times lower compared to the single-energy CT protocols. This difference in radiation dose derived from the fact that dose modulation was deactivated for all single-energy CT protocols. The results of this study show that an increase in radiation dose does not automatically reduce metal artifacts and that beam-hardening effects play a more important role than photon starvation in the formation of artifacts (Table 2**)**.

### Image quality at the bone/prosthesis interface

The dual-energy CT scan protocol 7 received the highest rating for image quality at that bone/prosthesis interface (1st rank), followed by the dual-energy CT scan protocols 6 (2nd rank) and 5 (3rd rank). Both single-energy CT protocols offered considerably lower scores (8th and 9th rank) (Table 2).

### Pairwise subjective evaluation of image quality

Image quality at the bone/prosthesis interface of the full titanium and ceramic prostheses (LCS^®^/NexGen^®^/Peter Brehm) was ranked superior compared to the conventional CoCr versions. All ceramic-coated prostheses (ACS^®^/Vanguard™/Columbus^®^/Scorpio^®^ and the Oxinium^®^ prosthesis) were ranked similar or slightly worse than the CoCr version (Table 3; Fig. 3**)**.

## Discussion

In this study, full titanium and ceramic prostheses provided significantly fewer artifacts on CT, compared to CoCr, and allowed for an excellent diagnostic assessment of the bone/prosthesis interface. In addition, the extent of metal artifacts can be significantly reduced with the use of mono-energetic image reconstructions of dual-energy CT imaging compared to conventional single-energy CT. CoCr steel models with a surface coating offer only slightly fewer blooming and streak artifacts compared to their corresponding CoCr steel alloy models, and the ‘subjective’ image quality was almost identical. The zirconium alloy prosthesis showed significantly less mean virtual growth, in contrast to considerably more streak artifacts than the corresponding standard CoCr model.

Although postoperative imaging of TKAs includes routine radiographic follow-ups [17], artifact-free CT visualization of TKAs plays an important role in the assessment of periprosthetic loosening or detection of periprosthetic osteolysis in cases with particle wear [21, 28]. In these cases, excellent image quality and a low artifact incidence are crucial. In particular, in posterior stabilized TKAs, a less attenuating material, such as titanium or ceramic, could be an advantage because the metal box of the posterior stabilized TKA blocks the visualization of the distal aspect of the condyles [15] and is associated with a higher amount of artifacts, as seen in the only posterior stabilized TKA (NexGen^®^) in this study.

To provide superior image quality of total knee arthroplasty of the bone/prosthesis interface, the utilization of titanium and ceramic as the main composite of TKA is supported by our results. However, today, the use of full titanium prostheses is limited by considerably higher costs, compared to the standard models.

With regard to the CT protocols, dual-energy CT with mono-energetic image reconstructions offered significantly fewer blooming and streak artifacts compared to single-energy CT. Even though the extent of artifacts could be lowered with these protocols, CoCr steel prostheses still showed major artifacts that most likely hamper the diagnostic ability to verify potential loosening on CT.

As expected, the higher tube current (mAs) used in single-energy CT protocols led to an overall smoother image with less noise in most parts of the CT image. However, even at very high tube currents, up to fourfold, the metal artifacts were not significantly reduced with the single-energy CT protocols, not at 120 kV, nor at 140 kV. This observation suggests that beam-hardening effects contribute a much greater proportion of the observed artifacts in the vicinity of implants than photon starvation effects.

Based on this assumption, it has to be noted that the dual-energy CT protocols that were applied in this study used a tin filter (selective photon shield, Siemens Medical Solutions, Forchheim, Germany) for the tube operating at 140 kV. Using a thin filter, the X-ray beam is already hardened, and is therefore, less susceptible to beam-hardening effects that might occur at the site of a metal prosthesis. Therefore, this tin filter might considerably contribute to the better image quality and the reduced amount of artifacts observed in the dual-energy CT reconstructions. In addition, mono-energetic image reconstructions of dual-energy CT scans have already proven their beneficial effect on artifact reduction near metal implants [1, 11]. However, in this study, we did not assess the proportions at which both techniques contribute to the observed amount of artifact reduction, thus emphasizing the need for further studies to assess whether single-energy CT with a tin filter might be comparable to dual-energy CT with regard to artifact reduction.

Several limitations are present in this study. All data were acquired in a phantom setup, and therefore, some clinical parameters, such as small movements of the patient, were not considered. However, the phantom setting used allowed for consistent results when comparing a variety of different CT protocols and prostheses without the dose restrictions that would apply for patients. In addition, the diagnostic accuracy for the detection of possible osteolysis at the bone/prosthesis interface that would indicate loosening was not examined. Instead, subjective image quality at the bone/prosthesis interface to determine the best scan parameters suitable for this region was assessed. Finally, different post-processing reconstruction methods, such as metal artifact reduction algorithms, were not compared.

With regard to day-to-day clinical work, the use of dual-energy CT is suggested by the results of this study to improve assessment of the bone/prosthesis interface in CT imaging of painful TKA. In addition, the material composition of TKA might be considered an important factor in TKA choice in the future.

## Conclusion

In conclusion, a TKA prosthesis that consisted of full ceramic and full titanium provided significantly fewer artifacts and a superior image quality for the bone/prosthesis interface compared to a standard CoCr alloy prosthesis. Dual-energy CT protocols with mono-energetic imaging provided a significantly better image quality, as well as fewer streak artifacts and blooming artifacts, with a lower radiation dose compared to single-energy CT.

## Electronic supplementary material

Below is the link to the electronic supplementary material.

Supplementary material 1 (DOCX 306 KB)

Supplementary material 2 (TIF 22785 KB)
