# Supplementary material for: Dual-energy CT and ceramic or titanium prostheses material reduce CT artifacts and provide superior image quality of total knee arthroplasty
Source: Knee Surg Sports Traumatol Arthrosc. 2018 Jun 7;27(5):1552–61. doi: 10.1007/s00167-018-5001-8 (PMC6527539; doi:10.1007/s00167-018-5001-8)
Supplement: Supplementary file 1 — Supplementary material 1 (DOCX 306 KB) [file 167_2018_5001_MOESM1_ESM.docx]

**Supplementary Material**

**Dual-energy CT image reconstruction**

After CT scanning, images were transferred to an offline workstation (Syngo Multi Modality Workplace [MMWP], Siemens AG, Healthcare Sector, Forchheim, Germany). The dual-energy CT datasets were post-processed using clinically available dedicated software (Syngo, Dual-Energy, MMWP version VE 36A) that was provided by the vendor to reconstruct mono-energetic images at a level of 105 keV, without changing slice thickness or reconstruction interval, as recommended by Bamberg et al. [1].

**Quantitative image analysis**

**Details of ROI measurements for streak artifacts**

The ROIs for quantitative measurements had a circular shape with a diameter more than twice as thick than most of the streak artifacts (10 mm diameter). In a normal setting without any streak artifacts, these regions of interest will offer homogeneous HU with a very low standard deviation, reflecting only the noise of the CT image in this area. In the presence of streak artifacts in an ROI that are thinner than that ROI itself, the standard deviation is highly elevated due to the presence of very low HU pixels compared to the expected HU of water or Play Doh^®^. For verification of this method, an additional ROI was measured in water and in Play Doh^®^ for each of the 144 scans above the level of the prosthesis, which was artifact-free. The position of the ROIs was standardized for all prostheses and scans, and is demonstrated in the figure below.

**Details of blooming artifact measurements**

The readers were blinded to the scan parameters, the material of the prosthesis, and the chosen order of the CT protocols which was different for each prosthesis during the readouts. Both the medial and lateral posterior femoral condyle of the TKA were measured by the readers, and, for the final analysis, the average of these two measurements was used for statistical analysis. In addition to the measured diameters of the condyles, the actual diameters of the condyles were measured with an electronic caliper (Lux-Tools, Wermelskirchen, Germany). All measurements for real diameter and blooming artifacts were measured in millimeters (mm).

**
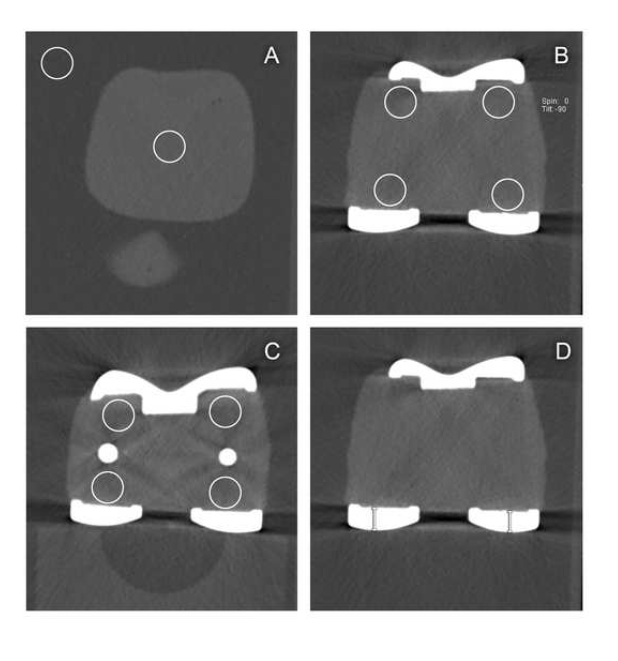
**

The figure shows the ROI (streak artifacts) and caliper positioning (blooming artifacts) on the LCS® TiNi prosthesis: ten ROI measurements were performed for each single prosthesis. One reference measurement in water and one measurement in the Play Doh above the level of the prosthesis (A) were performed. The location of the ROI was chosen in accord with the areas of clinical interest (bone/prosthesis interface). In total, eight measurements were performed near the prostheses at two different positions (B and C). The virtual growth (blooming) was measured at the maximum thickness of the posterior condyle of the femoral part of the prosthesis (D).

**Details of qualitative image analysis**

At one time, for each protocol of a particular prosthesis, one image was displayed on the four-panel PACS monitor. All simultaneously displayed images were obtained from the same table position and were, therefore, identical to each other except for the image quality, which was dependent on the scan protocol.
