# Supplementary figures and images for: Dual-energy CT and ceramic or titanium prostheses material reduce CT artifacts and provide superior image quality of total knee arthroplasty
Source: Knee Surg Sports Traumatol Arthrosc. 2018 Jun 7;27(5):1552–61. doi: 10.1007/s00167-018-5001-8 (PMC6527539; doi:10.1007/s00167-018-5001-8)

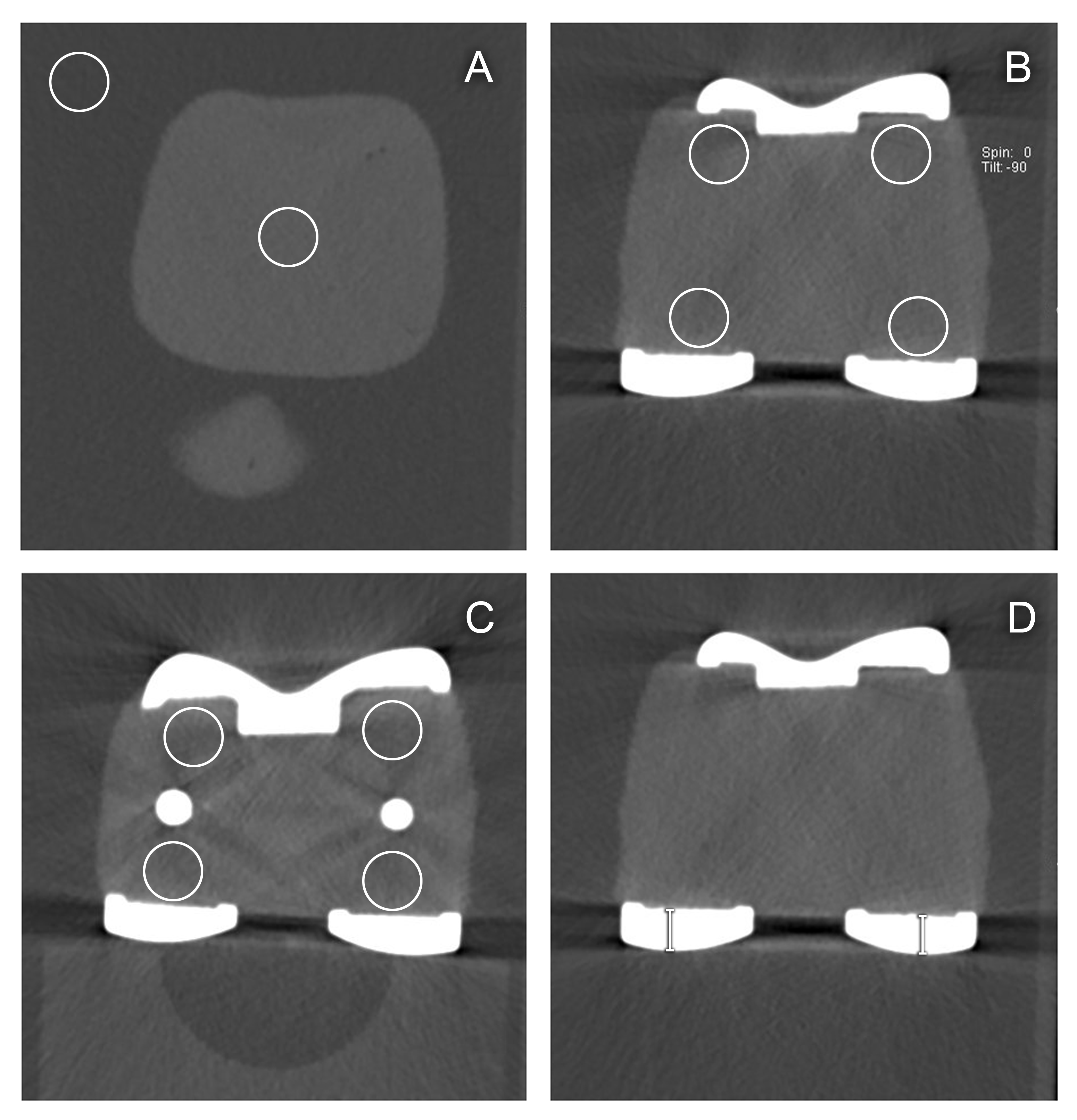

Supplement: Supplementary file 2 — Supplementary material 2 (TIF 22785 KB) [file 167_2018_5001_MOESM2_ESM.tif]
